# Modeling and Model Verification of the Stress-Strain State of Reinforced Polymer Concrete

**DOI:** 10.3390/ma16093494

**Published:** 2023-05-01

**Authors:** Kassym Yelemessov, Layla B. Sabirova, Nikita V. Martyushev, Boris V. Malozyomov, Gulnara B. Bakhmagambetova, Olga V. Atanova

**Affiliations:** 1 Institute of Energy and Mechanical Engineering, Satbayev University, Almaty KZ-050000, Kazakhstan; k.yelemessov@satbayev.university; 2 Department of Oil and Gas Production, Satbayev University, Almaty KZ-050000, Kazakhstan; slb2609@mail.ru; 3 Department of Advanced Technologies, Tomsk Polytechnic University, 634050 Tomsk, Russia; 4 Department of Electrotechnical Complexes, Novosibirsk State Technical University, 20, Karl Marks Ave., 630073 Novosibirsk, Russia; borisnovel@mail.ru; 5 Department of Mining, Satbayev University, Almaty KZ-050000, Kazakhstan; 6 Scientific Department, Satbayev University, Almaty KZ-050000, Kazakhstan

**Keywords:** geopolymer concrete, rubber polymer concretes, composite materials, concrete structures, strength analysis

## Abstract

This article considers the prospects of the application of building structures made of polymer concrete composites on the basis of strength analysis. The issues of application and structure of polymer-concrete mixtures are considered. Features of the stress-strain state of normal sections of polymer concrete beams are revealed. The dependence between the stresses and relative deformations of rubber polymer concretes and beams containing reinforcement frame and fiber reinforcement has been determined. The main direction of the study was the choice of ways to increase the strength characteristics of concrete with the addition of a polymer base and to increase the reliability of structures in general. The paper presents the results of experimental and mathematical studies of the stress-strain state and strength, as well as deflections of reinforced rubber-polymer beams. The peculiarities of fracture of reinforced rubber-polymer beams along their sections have been revealed according to the results of the experiment. The peculiarities of fracture formation of reinforced rubber-polymer beams have also been revealed. The conducted work has shown that the share of longitudinal reinforcement and the height of the fibrous reinforcement zone are the main factors. These reasons determine the characteristics of the strength of the beams and their resistance to destructive influences. The importance and scientific novelty of the work are the identified features of the stress-strain state of normal sections of rubber-concrete beams, namely, it has been established that the ultimate strength in axial compression and tension, deformations corresponding to the ultimate strength for rubber concrete exceed similar parameters for cement concrete 2.5–6.5 times. In the case of the addition of fiber reinforcement, this increase becomes, respectively, 3.0–7.5 times.

## 1. Introduction

Sustainable economic development of the countries in the world has become one of the main global environmental problems in many countries, and in the last decade, it has been paid much attention. There is an urgent need to develop appropriate strategies for the disposal of various types of waste such as plastic, tires, rubber, and glass. It is estimated that around 1.5 billion rubber tires are disposed of worldwide every year. The materials used in the production of tires are made up of complex mixtures. As a result, the share of discarded recycled tires is relatively low, with over 50% remaining unused.

Rubber industry waste and used tire rubber (UTR) is a non-degradable material that is not properly recycled. UTR also takes a significant amount of time to naturally degrade due to the cross-linked structure of the polymer material and additives such as stabilizers. Burning UTR releases toxic gases. Landfills and waste disposal cause serious environmental pollution of soil, water, and air. It pollutes the soil by killing beneficial bacteria and releasing toxic gases. Waste can be considered a potential resource and valuable material. A logical way to reduce the negative impact of rubber waste and its cost is to use it in construction and industry. Natural aggregates are more valuable than waste. When looking for economically viable deposits to extract non-metallic materials, factors such as transportation costs, the quality of the aggregate suitable for mining, government regulation, and the cost of operating and maintaining vehicles are taken into account. At the same time, transport costs for aggregates are very high. In addition, the energy required to crush the rock into aggregates is proportional to the area of the new surface, and therefore it constitutes a significant part of the energy consumed in the production of aggregates [1]. UTR easily absorb energy and have excellent sound and heat insulation properties, making them suitable for use in a variety of applications screens, and asphalt concrete mixtures [1,2]. Over the past 20 years, researchers have studied the possibility of using UTR as an aggregate in polymer concrete mixes. The use of rubber additives in concrete structures provides concrete products with new properties, including strength, reliability, and resistance to aggressive environments.

Polymer concretes are widely used in the construction industry and other industries. To date, a large amount of experimental data on the study of their structure and properties has been accumulated [1]. At the same time, despite a sufficiently large number of already conducted studies, there are difficulties in selecting optimal compositions for manufacturing the structures. Insufficient elaboration and studying the law of the structure’s formation and properties of polymer concrete create complexities in their application [2]. The establishment of such regularities is an extremely difficult task.

Polymer concretes are a type of polymer composite material. The main area of application of such composites is construction. In modern representation, polymer composites are a rather complex hierarchical system formed as a result of physical and chemical interactions between its structural components [3]. The main feature of composites is their ability to form specific structures responsible for the acquisition of non-additive, sometimes unique properties of the composite. Such structures may include fractal, cluster, and lattice structures, the analysis of which is paid more and more attention in modern construction material science [4]. Properties of polymer concrete at the microstructure level are determined by the phenomena occurring during the contact of liquid and solid phases, i.e., they depend on the amount of filler, its dispersity, and physical and chemical activity. There is no universal optimal filler content for composites. Depending on the conditions of application of polymer concretes, this value can take different values. Usually, the optimal content of the filler provides the highest performance indicators of polymer concretes [5]. In this regard, the use of fillers having discontinuous granulometry, i.e., having different geometric fractions, is effective. The study of the influence of aggregates and their role in the structure formation of polymer concretes is an important problem considered in this article.

In this paper, the most promising polystructural theory of construction composites is taken as the basis. It is based on the concept that consists of the representation of construction composites as polystructured. They are composed of many structures, passing one into another according to the “structure-in-structure” principle, in which there are fractions of additional composite materials of several sizes (powder and granules) [6]. There is an organic connection between the structures of different levels and sublevels. The formation of structures of a higher scale level occurs under the influence of structures of a lower level. At the same time, the higher-level structures may determine the substructure formation conditions by the feedback principle. At present, based on the results of numerous practical and theoretical studies, ideas about the optimal structure of construction composites as a matrix medium with dispersed particles evenly distributed in it are being revised [7]. The practical unattainability of such an “ideal” structural situation has been established. On the contrary, during the technological processes of preparation and the curing of construction composites, the structural components of composites tend to combine various kinds of heterogeneities, differently affecting the properties of composites. It is possible to change the structure of construction composites by changing the formulation and technological factors. New structural heterogeneities can be introduced, or existing structural heterogeneities can be changed at various structural levels. The formation of boundary transition layers of the polymer matrix in polymer concretes has a direct impact on their performance properties (flexibility, elastic deformations, cracking resistance). Therefore, the analysis of their formation requires a more detailed consideration. The recommended methods of selecting the particle size distribution of aggregates are difficult to implement and do not guarantee to obtain the smallest intergranular hollowness, since they do not consider the properties of individual fractions of aggregates and their mutual distribution. The properties of polymer concretes depend not only on the quality of the initial components and their mutual arrangement but also on the nature of the interaction between them [8]. To create high-quality polymer concretes, it is necessary to have a strong, chemically and thermally, stable bond between the surface of aggregates and the polymer matrix. The considered modern methods of predicting the properties and calculation of compositions of polymer concretes are almost all based on the selection of the mineral mixture with the lowest hollowness by the method used for cement concrete. Recently, the methods for predicting the properties and calculating compositions of composite materials of reduced polymer capacity, provided that there is a given set of properties based on the models of different types obtained as a result of research, have become widely used. This depends not only on the material properties, but also on the technology of its production and, most importantly, on the purpose and application of polymer concrete structures [9].

To ensure a reliable efficient operation of elements made of new types of concrete, it is necessary to study the stress-strain state arising under the action of forces of various kinds, in particular, a bending moment. In this connection, the study of resistance to the bending moment action of normal sections of beams made of reinforced polymer concrete (ACRP) (beams containing a reinforcement frame and fiber reinforcement located at different heights of the section relative to the bottom edge) is of scientific interest and a practical important research task.

The conducted research analysis of force resistance of polymer concrete and reinforced concrete bendable elements has shown that the application of polymer concretes and structures on their basis is actually because of their inherent high operational characteristics. It is important to study the degree of influence of dispersed reinforcement on the performance characteristics of polymer concrete structures. The main methods existing today for polymer concrete structures’ calculation are quite limited in terms of the used materials. Their main scope is structures made of furfural acetone concrete, polyester, and epoxy concrete. This is due to the existing wide experimental base containing these materials and a large number of obtained empirical dependences [10].

In this regard, to improve the strength of polymer concretes and the reliability of structures, as well as to predict the properties of the material and products made of it, the following tasks were set in this article:

- to carry out an analysis of force-resistance studies of polymer concrete and reinforced concrete beams;
- to evaluate the physical conditions of polymer concrete structures based on rubber additives;
- to conduct experimental studies of the stress-strain state and strength and deflections of ACRP beams;
- to reveal the peculiarities of failure of ACRP beams by their cross-sections;
- to reveal the peculiarities of the formation of the failure of ACRP beams;
- to reveal the peculiarities of the development of deflections of ACRP beams.

## 2. Methods and Materials

There is a sufficiently large production and waste volume of synthetic rubber and rubber products in modern industry [2,3]. Their use as a binder in the production of polymer concrete is therefore of practical interest. They have a low cost in comparison with that of used polymer resins. It is also possible to use liquid rubbers having specified properties. However, in this work, rubber was chosen as a binder. It is important to note that the production of polymer concretes based on rubbers due to the high filling of the mixture can reduce the cost of the composite [11,12].

### 2.1. Rationale for the Material Choice

Based on previous studies, the optimum ratios of hardening group components containing a binder, aggregate, and filler were determined [13]. It was found that the introduction of fly ash, which is a hard-to-dispose waste, into the composition of rubber polymer concrete (RPC), has a positive effect on its strength and chemical properties. Moreover, in these works, studies were conducted to study the chemical resistance of polymer concrete and it was found that polymer concrete has almost universal chemical resistance to various inherently aggressive environments.

Based on the analysis of literary sources and in the course of experimental studies, the authors obtained a comparative table (Table 1) of polymer concretes based on rubbers, which have the potential to be used for the manufacture of structures [2,3,6,7,12]. The main characteristics of rubber concrete used in the work are presented in Table 1.

In practice, the property that determines the scope of polymer concrete is heat resistance at 80–150 °C. At the same time, when the operating temperature of polymer concrete increases, its strength, and modulus of elasticity decrease. Having low heat resistance, polymer concretes nevertheless belong to the class of non-combustible materials, since the content of organic matter in them is low as compared to the proportion of inorganic components.

Since a promising direction in the study of polymer concretes is to reinforce them with dispersed reinforcement, additional reinforcement was used in the fabrication of specimens [14]. As a result, the beam specimens were made of reinforced rubber fiber reinforced concrete (ACPBF). The dispersion reinforcement fiber was made from tire industry waste metal cord. A chaotic orientation of fibers along the volume of the element was used. It allows perceiving and redistributing efforts of different orientations, appearing in the sample, thereby preventing the appearance and development of cracks. It should also be noted that, for a tensile element, the failure of specimens occurs either during fibers rupture or when violating their adhesion to the RPC [15].

### 2.2. Calculation of Polymer Concrete Structures

**Figure 1:**
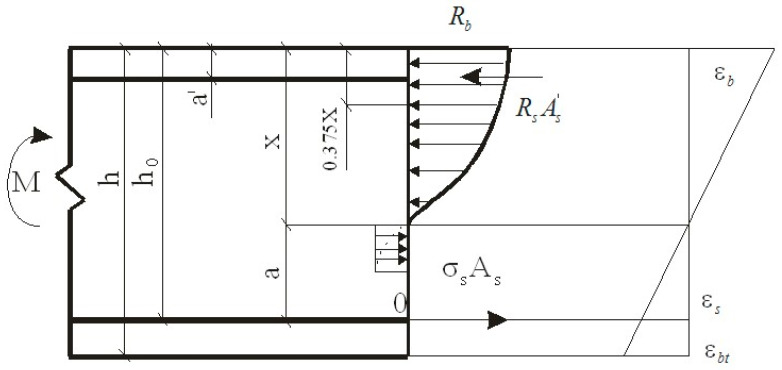
The scheme of forces and the stress diagram in the section normal to the longitudinal axis of the bendable polymer concrete element, when calculating its strength.

The basis of the methodology for calculating polymer concrete structures is set out in [16]. The strength calculation methodology is based on:

1. normal stresses, corresponding in shape to the diagram of mechanical tests for axial compression and tension of polymer concrete;
2. the hypothesis of planar sections [16].
3. Three equations were made by the authors to obtain calculation formulas:
4. moments of forces equilibrium;
5. projections of forces on the neutral plane equilibrium;
6. equations of a ratio of boundary deformations or heights of compressed and stretched zones of the element cross-section. The scheme of forces and the stress diagram in the cross-section normal to the longitudinal axis of the bendable polymer concrete element, when calculating its strength, is shown in Figure 1.

First of all, let us write an equation for the moments of forces equilibrium.

When reinforcing the tensile and compressed zones of the bendable polymer concrete element (Figure 1), the calculation formula is as follows:

(1) $$M\leq \sigma_{s}\cdot A_{s}(h_{0}-0.375\cdot x)+R_{s}\cdot A_{s}\prime (0.375\cdot x-a^{\prime })$$

where $a^{\prime }$ represents the distance between an adjacent compression-deformed fiber and the axis of the compression reinforcement;

*a* is a tension region height;

x is the height of the compressed zone, determined by the formula:

*M* is a bending of the beam during tests;

*R_s_* is a design resistance of the longitudinal bar;

*A_s_* is an area of reinforcement in the tension region;

*A’_s_* is an area of reinforcement in the compression region;

ξ is a relative height of the compression region of the cross-section determined by the formula [17]:

(2) $$\xi =\frac{x}{h_{0}}=1.5\frac{R_{s}}{R_{compr}}(\mu -\mu^{\prime }).$$

where *µ* is a reinforcement ratio in the tension region;

*µ’* is a reinforcement ratio in the compression region;

*R_compr_* is a design resistance in the compression region.

The calculation procedure [17] for the formation of cracks normal to the longitudinal axis of the element is based on a rectangular stress diagram in the tensile zone and a triangular diagram in the compressed zone at the height of the tensile zone a, equal to:

(3) $$a=h_{0}-x.$$

Let us express *x* based on Formula (3) and use it in Formula (2). Let us also express $\left(\mu -\mu^{\prime }\right)$ using the same formula, transferring the values $1.5\frac{R_{s}}{R_{compr}}$ to the opposite part of the formula. Then, the difference in reinforcement coefficients in the tensile and compressed zones $\left(\mu -\mu^{\prime }\right)$ is determined by the formula:

(4) $$(\mu -\mu^{\prime })=\frac{0.67\frac{R_{compr}}{R_{s}}}{1+\frac{E}{E_{s}}\cdot \frac{R_{s}}{R_{compr}}}.$$

According to [18], the stresses in the longitudinal reinforcement $\left(\sigma_{s}\right)$ do not reach the yield strength. Let us substitute expression 3 for expression 1 and convert decimals to natural fractions. When converting, the following facts must be considered. To use polymer concrete most completely, it is advisable to provide a clearly defined curved shape of the stress epure in the compression cross-section region, which requires a rather high percentage of reinforcement of about 15% or more. In the case of lower reinforcement percentages, the triangular shape of the epure is to be used. Therefore, the calculation of crack formation is based on a rectangular stress epure in the tension region and a triangular one in the compression region at the height of the tension region. Proceeding from this fact, the bending moment before the formation of cracks is determined by the formula:

(5) $$M\leq \sigma_{s}\cdot A_{s}(h_{0}-\frac{x}{3})+R_{s}\cdot b\frac{3\cdot h+x}{6}(h-x).$$

Stresses in the longitudinal reinforcement $\left(\sigma_{s}\right)$ are found owing to the deformation of the reinforcement $\left(\varepsilon_{s}\right)$ and polymer concrete at the reinforcement level of:

(6) $$\sigma_{s}=\varepsilon_{s}\cdot E_{s}.$$

The reinforcement deformations are equal to:

(7) $$(\varepsilon_{s})=\varepsilon_{bt}\frac{h_{0}-x}{h-x},$$

where $\varepsilon_{bt}$ is the deformation of the lower tensile edge of the bendable polymer concrete element. It follows from the hypothesis of flat sections that:

(8) $$\frac{\varepsilon_{b}}{x}=\frac{\sigma_{b}}{E_{b}\cdot x}=\frac{\varepsilon_{s}}{h_{0}-x}=\frac{\varepsilon_{bt}}{h-x},$$

where $\varepsilon_{b}$ is the deformation of the upper compressed face of the bendable polymer concrete element;

$\sigma_{b}$ is stresses in the upper compressed face of the bendable polymer concrete element. Based on this, we determine the $\beta^{\prime }$ as the height of the compressed zone:

(9) $$\beta^{\prime }=\frac{\varepsilon_{s}}{\varepsilon_{b}}=\frac{\varepsilon_{s}\cdot E_{b}}{\sigma_{b}}.$$

In the first case, the height of the compressed zone is expressed through the deformation of the reinforcement:

(10) $$\beta^{\prime }=\frac{\varepsilon_{s}}{\varepsilon_{b}}=\frac{\varepsilon_{s}\cdot E_{b}}{\sigma_{b}}.$$

On the other hand, it is possible to make through the deformation of the outermost stretched fiber, that is, through the ultimate tensile strength:

(11) $$\beta =\frac{\varepsilon_{bt}}{\varepsilon_{b}}=\frac{\varepsilon_{bt}\cdot E_{b}}{\sigma_{b}}.$$

By projecting the forces onto the neutral plane, we obtain:

(12) $$\sigma_{s}\cdot A_{s}+R_{bt}\cdot b(h-x)=0.5\cdot \sigma_{b}\cdot b\cdot x.$$

The reinforcement factor is determined by the formula:

(13) $$\mu =\frac{A_{s}}{b\cdot h_{0}}=\frac{0.5\cdot x-R_{bt}(h-x)}{\sigma_{s}\cdot h_{0}}.$$

The compressive edge stresses $\left(\sigma_{b}\right)$ are set from the calculation so that they do not go beyond the linear section of the diagram, i.e., “σ−ε”$\sigma_{b}<0.75R$ [19]. Let us substitute the obtained Formula (6) into expression 5. Let us also consider the fact that the calculation formula for the bending moment, when reinforcing the compression region of the beams before the formation of cracks, will receive an additional summand. The formula for determining the moment will take the following form:

(14) $$M=\varepsilon_{s}\cdot E_{s}\cdot A_{S}(h_{0}-\frac{x}{3})+R_{s}\cdot b\frac{3\cdot h+x}{6}(h-x)+\sigma_{s}^{\prime }\cdot A_{s}^{\prime }(\frac{x}{3}-a^{\prime }),$$

where $\sigma_{s}^{\prime }$ is stress in the compressed reinforcement, determined by the formula:

(15) $$\sigma_{s}^{\prime }=\sigma_{b}\frac{E_{s}}{E_{b}}\cdot \frac{x-a^{\prime }}{x}.$$

### 2.3. Experimental Design and Research Program

BHP has a high tensile strength [19], as well as a higher ultimate tensile strength, compared to cement concrete. These properties can be effectively used to increase the crack resistance of bendable elements. In addition, if the fiber is added to the bent element in this way, the dispersion increases in the height of the cross-section (partial or full). An element or a two-layer structure having a fibrous reinforcement in the stretching zone is obtained, whose function will increase even more [20]. To achieve this goal, we decided to determine the normal cross-section of the ACPBF bending element, having the performance characteristics of dispersed steel bars over the entire height of the cross-section, and to obtain data on its tensile strength, resistance to cracking, and deformability [21].

The testing of beams made of rubber concrete involved test specimens-beams of all series made having a section size of 6 × 12 cm and a total length of 140 cm. The beams were manufactured according to the Central Asian standards, GOST 948-2016 (ICS 91.080.40), the Interstate Standard of Reinforced-Concrete, and Polymer-Concrete Lintels. The determining parameters of the beams are the following: the length (L) is from 1030 (mm) to 5950 (mm); the width (B) is from 120 (mm) to 250 (mm); the height (H) is from 60 (mm) to 585 (mm). The beams are tested by means of two symmetrically applied forces in the thirds of the span (pure bending test procedure). In this way, the samples of the beam were made of reinforced rubber fiber concrete. The dispersion reinforcing fiber was made of waste metal cord from the tire industry. Various parameters are assigned in the experiment:

- the percentage of the longitudinal gain that has the greatest effect on the resistance of the normal part bending element;
- the height of the dispersion reinforcement zone (measured from the bottom edge) with the same percentage of fiber reinforcement over the volume of the reinforced part (µv = 1%, according to composition optimization [22]).

The response function is the strength; they combine the crack resistance of an element of the normal cross-section, as well as their good deformability. The boundary conditions of the area of variation of the experimental parameters are established on the basis of the analysis of the literature [23]. The procedure for testing beams complies with the Central Asian Standards GOST 10180-2012 and ICS 91.100.30, Methods for Strength Determination Using Reference Specimens. The percentage of longitudinal reinforcement gradually increases from 0% (no longitudinal reinforcement) to 8.4% (compression zone failure). The height of the dispersed reinforcement zone is determined based on the testing of AKPBF samples, which are reinforced along the entire length. To determine the physical and mechanical properties of the material used for the manufacture of the test beam, together with the beam, a control sample of a 4 cm × 4 cm × 16 cm prism is made and tested for compression, and a control 8 cm × 4 cm × 40 cm sample is tested for tension.

**Figure 2:**
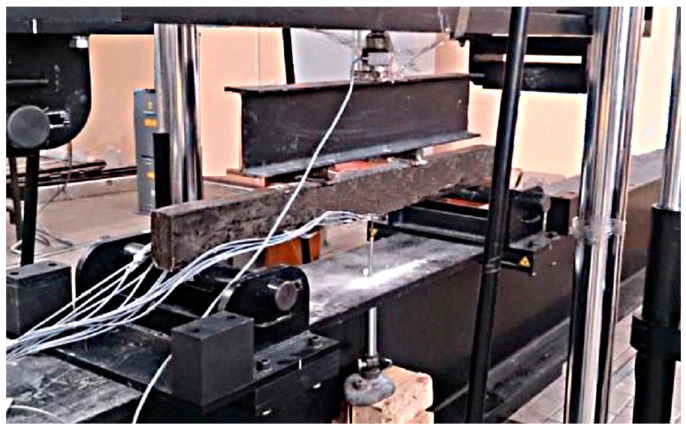
General view of the bending test of rubber concrete beams.

The test procedure complies with GOST 10180-2012 and ICS 91.100.30, Methods for Strength Determination Using Reference Specifications. In the laboratory, before testing the samples, the temperature is maintained at 20 ± 5 °C and the relative humidity of the air is at least 55%. Under these conditions, the samples were in the dismantle form before the test for at least 24 h. The control samples were tested on the day of testing the beam. The beams were loaded with two equal concentrated loads, applied vertically in the thirds of the span. In the case of this type of load application, the value of the bending moment, arising in the beam, increases from zero on the support to the maximum value under the point of load application. Between the points of load application, the transverse force is zero, and the value of the bending moment is constant and equal to the maximum value (the pure bending zone). A general view of testing the samples of rubber concrete beams is shown in Figure 2.

The beam samples were tested using a laboratory press “INSTRON 600KN” (60 tons), certified and meeting the requirements of GOST 10180-2012 and ICS 91.100.30 “Methods for Strength Determination Using Reference Specifications”. The load on the sample was fed at a constant rate until destruction.

### 2.4. Materials

The following materials were used in the work:

- Cis-polybutadiene low molecular weight CBLMW-R rubber (ISO 6743/4). CBLMW-R rubber has a density of 910 kg/m^3^ and a dynamic viscosity of up to 12 Pa × s;
- fine-dispersed filler being ash of a specific surface of 2500...2700 cm^2^/g, having the following composition by mass in %: Al O_23_—16.5–22.5; CaO was 5.5–5.5; Fe O_23_ was 13.5–15.5; SiO_2_ was 47–55; MgO was 2–3; K_2_ O was 1–2; Na_2_ O was 1; S O_23_ was 0.4–0.3; others were 6–15;
- vulcanization activator was zinc oxide ZnO (ISO 10262-2016) white powder having a density of 5600–5700 kg/m^3^;
- vulcanization gas pedal was tetramethyl thiuram disulfide (Tiuram-D) (ISO 4097 2013) being powder of gray-white color and density of 1300–1400 kg/m^3^;
- calcium oxide CaO was a white powder having a density of 2500–2900 kg/m^3^;
- Portland cement. It consists of the following ingredients: clinker (calcium silicates); gypsum; plasticizing, hydrophobic, acid-resistant additives; domain fee. The chemical composition of cement: 21.55% silicon oxide and 65.91% calcium oxide. Portland cement is the most durable and high quality, therefore it is widely distributed on the market. It is a cement that is capable of showing an average compressive strength of about 500 kg/m^3^ after 28 days of preparation. The mixture can be without additives or with various substances introduced into the composition in a certain proportion. The compressive load is 2500 kg/cm. Frost resistance is more than 100 cycles. The bulk density is 1100–1600 kg/m. True density kg/m^3^ 3100. The setting speed is from 45 min to 10 h. The average weight of Portland cement is 3 tons/m^3^;
- sulfur technical (ISO 3704-76) is a bright yellow powder with a concentration of 2070 kg/m^3^, having a melting point of 114 °C;
- metal fibers made from scraps of metal cord by sawing. The fibers obtained in this way are wave-shaped fibers at a ratio of a length to a wire diameter of 1/100;
- sand and crushed granite [24] are selected in accordance with the relevant requirements of GOST 26633-2015. Concrete is heavy and fine-grained. Specifications and ISS 91.100.30. The physical properties of gravel and sand are given in Table 2.
- reinforcing steel bars having a diameter of 8, 10, 12, 14, 16, and 18 mm and a steel wire of a diameter of 5 mm.

### 2.5. Mechanical Tests

Determination of the actual physical and mechanical characteristics of steel reinforcement was carried out using the Instron 1500HDX testing system. Measuring the longitudinal parameter with 270 uses a base of a 20-mm strain gauge for deformation. A set of equipment was used to control the stress-strain state (VAR) stage, namely, deformation 2 MG plus design and CATMAN-AP software [25]. The control specimens (3 pieces of twin specimens) were tested for tensile strength before rupture according to the requirements [26].

The component composition of rubber concrete (as a percentage of the element weight) supplemented with dispersed reinforcement, which provides the best strength characteristics and chemical resistance, is shown in Table 3. These compositions were obtained as a result of optimizing the component composition of PBC [27] and are pleasant for the basic ones.

To manufacture concrete specimens without the filler, the concrete of a composition having a similar modulus of elasticity to that of rubber concretes was chosen. The composition of the cement concrete used for making experimental specimens without the filler (mass per 1 m^3^ in tons) [28] was as follows: sand—0.692; water—0.19; cement—0.364; crushed stone—0.121.

### 2.6. Sample Production Technology

The production of the elements made of fiber-reinforced polymer concrete (FRPP) with the arrangement of fibers along the entire section height (chaotically) was carried out in one stage.

Based on [29], the preparation of the FCPB mixture included the following operations:

- washing the fine and coarse aggregate;
- preparation of curing group components and fibers;
- dosage of ingredients;
- drying of the components;
- mixing of the components.

Dosing of sand and gravel was carried out on the scales VPS-40 M having an accuracy of 5 g. The dosing of other components was carried out on the electronic scales CAS ER JR-15CB having an accuracy of 1 g due to their smaller weight [30].

The components were mixed in a high-speed propeller-type mixer. The polymeric binder was prepared by combining CBLMW-R rubber with hardening group components and the fine filler with fly ash. The mixing time of the binder was 80 s at 1000 rpm. Then fine aggregate and fibers mixed with the coarse aggregate were introduced into the prepared mixture. The resulting polymer concrete mixture was prepared in the same mixer at a speed of 180 rpm for 200 s.

Before laying the FCPB mixture in the form, the surface is smeared with waste oil to facilitate removal later from the formwork. The prepared mixture was placed in the molds and compacted on the vibrating table with a vibration duration of 100 ± 30 s. An indication of sufficient compaction of the polymer concrete mixture is the release of the binder on the surface and the cessation of the intensive formation of air bubbles [31].

After performing the above operations, the mold with the mixture was placed in a dry heating chamber (dryer), where FKPB was cured at 115 ± 5 °C for 12 h (taking into account the heating time of 17 h). Unpacking was performed after the complete polymerization and cooling of the samples.

Fabrication of FKPB elements with the fiber arrangement in the tensile zone (chaotically) was carried out in two similar stages according to the works [32].

The fabrication of FCPB elements took place in one step using metal cord fibers.

**Figure 3:**
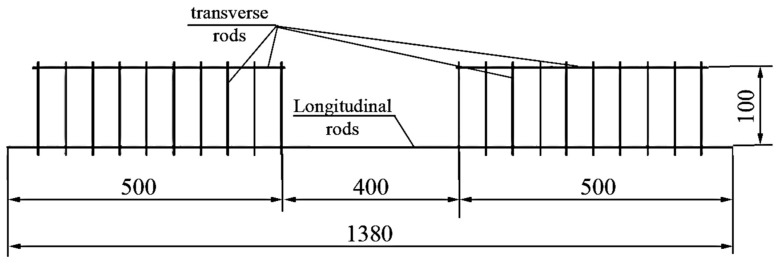
The reinforcement frame of the experimental beams. Dimensions are in mm.

The reinforcement of all series of experimental beams was a welded frame, shown in Figure 3.

Figure 2 shows longitudinal rods are reinforcement, transverse is the wire (a diameter of 4.5–5.5 mm), and the step of transverse bars is 50 mm in order to prevent fracture in the sloping sections of the experimental beams.

### 2.7. Test Methods, Basic Instruments and Equipment

The test procedure corresponds to the methods outlined in the regulatory literature [33].

The test of the manufactured beams was carried out using concentrated loads. The load was applied vertically and distributed in the third part of the beams’ span. In the case of a loading scheme for beams, the bending moment arising in the beams is 0 on the support. Already under the point of application of the load, the moment increases to a maximum value. Between the points to which the load is applied, the value of the transverse force is zero. The bending moment at the same point has a constant value equal to the maximum value. A diagram of the load distribution on the test specimens is shown in Figure 3.

**Figure 4:**
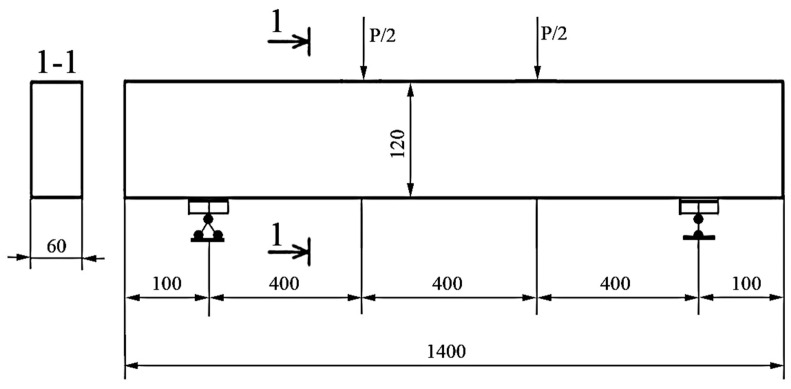
Load diagram and geometric dimensions of beams (unit: mm).

The beam samples were tested on the laboratory presses of INSTRON800KN (80 tons), certified, and they met the requirements [34]. The load was applied to the sample at a constant rate until it was destroyed (Figure 4).

The maximum value of the pressing force was the value of the breaking load during beam testing. It was determined by a force sensor. At this value, the yield strength of the reinforcement was reached. Glued strain gauges made it possible to measure longitudinal deformations. Deformation measurements were carried out in a normal section. The strain gauge base was 2 cm. A linear displacement transducer, a plunger, with an accuracy class of 0.2%, was used to measure the vertical displacements of the beams.

During the given tests, the crack opening width and height were measured. The crack opening width was measured with a micrometer; a duplicate measurement was made with a caliper.

Before testing, the specimens were inspected and measured. Roughness and burrs on the surface of the beam material were removed with an angle grinder. The side surfaces of the beams were ground to facilitate visual observation of the appearance and distribution of cracks due to the dark surface of the material. Before attaching the load cells, the surface of the beam was ground with an angle grinder and degreased with acetone. After that, the load cells were pressed to the surface of the structure with glue.

With each polymer-concrete beam, control samples-prisms (4 cm × 4 cm × 16 cm) and specimens-eights with the size of a working area of 3 cm × 4 cm and a total length of 40 cm were made. To control the deformation-strength characteristics, control samples-cubes (10 cm × 10 cm × 10 cm) and prisms (4 cm × 4 cm × 16 cm) were made with each concrete beam. Three control samples were produced. Studies of prisms and cubes were carried out under central compression and octagonal samples were tested under central tension.

Compression tests of beams, concrete prisms, and concrete cubes were performed according to the requirements [35] on an INSTRON Satec 1200 press (Instron Corporation, New-York, NY, USA) certified and meeting the requirements [36]. Strain gauges were installed on the specimens-prisms on the opposite faces to measure the longitudinal relative deformations arising in the specimen at a constant rate of 60 MPa/min when a short-term compressive load was applied. Before testing, the surface of the specimens was prepared and checked for the absence of defects (cracks, cavities, etc.), and the ends of the specimens were checked to be perpendicular to the longitudinal axis.

Tensile tests of fiber-reinforced polymer concrete (FRPB) specimens were carried out taking into account the requirements of ISO 1920-1:2004 and recommendations on testing methods for polymer concretes [37] on a tensile testing machine INSTRON 5982 (Instron Corporation, New-York, NY, USA), being certified and meeting the requirements [38]. Strain gauges were installed on the eight specimens on the opposite faces to take longitudinal relative deformations. Before testing, the specimens were also carefully prepared and checked for defects. The load was applied uniformly, continuously at a constant rate of 0.15 MPa/s.

## 3. Discussion

### 3.1. Results of Experimental Studies of Intensity (Construction of a Material Deformation Diagram “σ-ε”)

**Figure 5:**
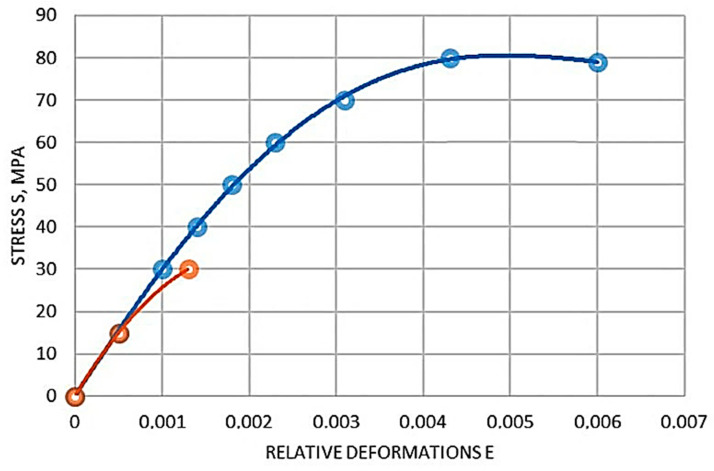
Diagram of the relationship between compressive stresses and relative deformations of FKPB products: red curve—y = −9 × 10^6^ x^2^ + 34,327 x (R^2^ = 1), blue curve—y = 6 × 10^10^ x^4^ − 6 × 10^8^ x^3^ − 2 × 10^6^ x^2^ + 32,199 x (R^2^ = 0.9996).

**Figure 6:**
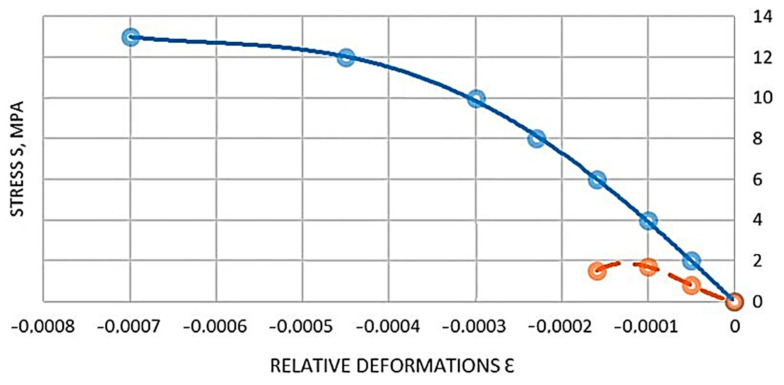
Diagram of the relationship between tensile stresses and relative deformations of FKPB products: red curve—y = 10^12^ x^3^ + 2 × 10^8^ x^2^ − 8314.4 x (R^2^ = 1), blue curve—y = 8 × 10^13^ x^4^ + 9 × 10^3^ x^3^ – 4 × 10^6^ x^2^ − 40,448 x (R^2^ = 0.9997).

In order to determine the relationship between stress and relative deformation occurring in FCPB, required for a comprehensive study of the cross-section of the deflection of a curved element, a control sample being a prism and a sample, eight were made from each experimental beam. Test methods of specimens, their geometrical parameters, and schemes of measuring instruments arrangement are given in Section 2.6. On the basis of the compression tests of prism specimens and tension specimens of octahedrons, graphs between the stresses and relative deformations of compression and tension for FKPB were obtained, these graphs are shown in Figure 5 and Figure 6, respectively.

Based on the diagram shown in Figure 5 and Figure 6, equations describing the relationship between stresses and relative strains were derived for the FCPB.

The relationship between compressive stresses and relative strains is shown below.

Based on the analysis of Figure 5 and Figure 6, equations describing the relationship between stresses and relative strains were derived for FKPB products.

The dependencies in the analytical form between compressive stresses and relative deformations of FKPB products are presented in Figure 6. The dependences between the tensile stresses and relative deformations of the FCPB products are shown in Figure 6.

### 3.2. Strength of Normal Sections

**Figure 7:**
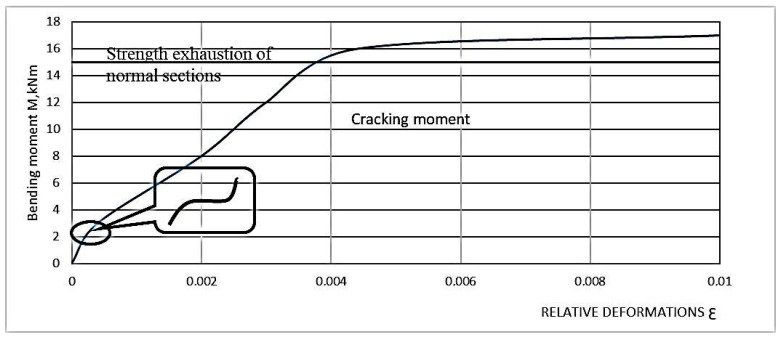
Relative tensile strains in the reinforcement of the FPB beam as a function of bending moments.

**Figure 8:**
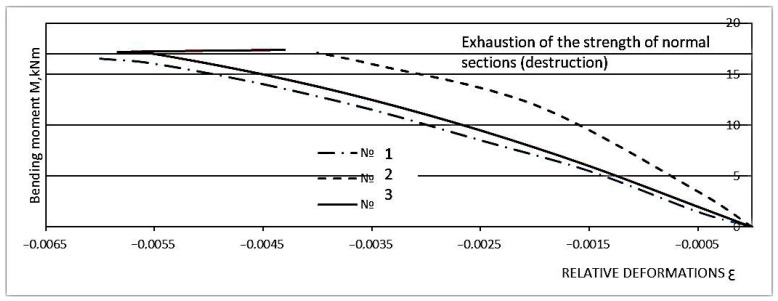
Relative deformations of the compressed zone of FPB as a function of bending moments. Nos. 1–3 is load cell numbers.

Experimental studies have shown that the percentage of longitudinal reinforcement and the height of the diffuse reinforcement zone are the main factors affecting the normal cross-sectional strength of the ACPB bending element. The parameters and test procedures of the test sample in the study of strength in normal cross-section are given in Section 2.5 and Section 2.6. The destructive bending moment (in case of fracture in the tensile zone) is referred to the moment when the stress in the steel rod reaches the yield point, which also corresponds to a stronger increase in the deformation in the steel rod (Figure 7 and Figure 8). The destructive bending moment (in case of destruction of the compression zone) is the moment when the compression zone collapses, which corresponds to a sharp increase in deformation. In this case, the curve corresponding to the probe deformations has a kink, and the deformations begin to decrease, which indicates the buckling in this area.

**Figure 9:**
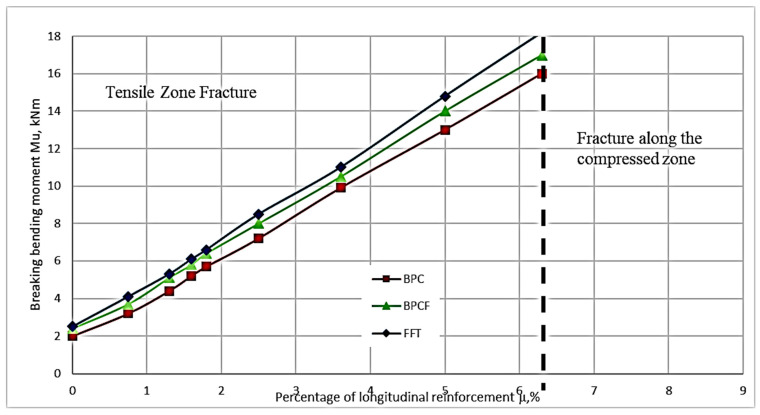
Diagram of the dependence of the value of destructive bending moments on the percentage of longitudinal reinforcement.

**Figure 10:**
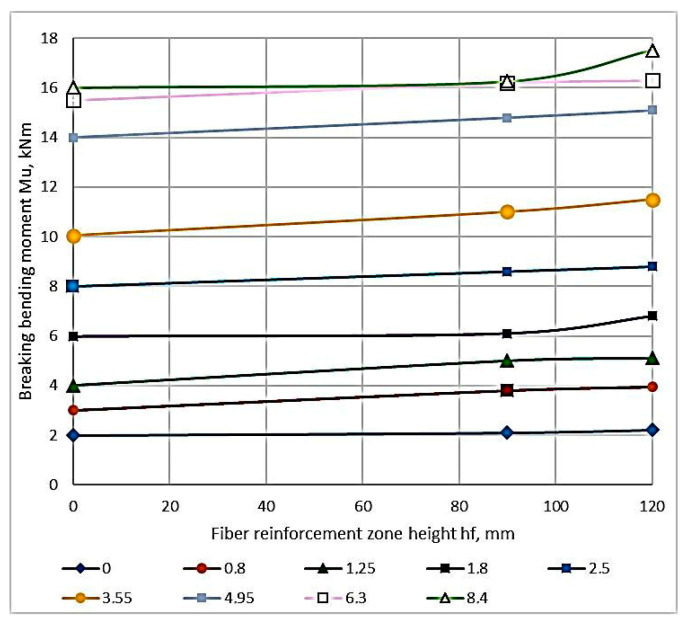
Dependence plot of fracture bending moments on the fiber reinforcement zone height.

As a result of the experimental studies, the values of the breaking bending moments depending on the percentage of longitudinal reinforcement and the height of the fiber reinforcement zone were obtained. These are presented in Appendix A. Based on the data in Table A1 (Appendix A.1), the dependence of the bending moment on each varying factor is plotted graphically (Figure 9 and Figure 10). Figure 10 shows a graph of the dependence of the destructive bending moment occurring in beams with the same rebar content on the height of the fiber reinforcement layer.

The study of the composition in Figure 9 and Figure 10 shows that the proportion of the filling of the longitudinal reinforcement has a significant effect. The reinforcement percentage affects the value of the breaking load more strongly than other factors do. The next parameter in terms of influence is the height of the fiber reinforcement zone. The height’s influence on the fiber reinforcement zone is significantly less than the reinforcement percentage influence.

The study of the composition of Figure 9 and Figure 10 confirms that the proportion of the longitudinal reinforcement content has a significant effect. The second varying parameter in the form of the height of the fiber reinforcement zone has an effect on the bearing capacity, but its influence is much smaller than that of the reinforcement longitudinal percentage.

In the range of longitudinal reinforcement percentage values of 0.6–6.5%, the dependence in Figure 9 is linear. An increase in the proportion of the presence of the longitudinal reinforcement in ACPBF beams leads to their destruction in the compression zone. However, in this case, there is no increase in strength characteristics for normalized sections.

The linear dependence between the parameters of the percentage of longitudinal reinforcement and the bending moment is maintained over a sufficiently large segment. This is a segment of the values of the percentage of longitudinal reinforcement equal to 0.8–6.3%. A further increase in the percentage of longitudinal reinforcement leads to a change in the nature of the destruction. The destruction of ACPBF beams occurs already in the compressed zone. The strength of the sections almost does not change in the presence of such destruction. It is important to note that the failure of ACPBF beams having a percentage of longitudinal reinforcement equal to 0.8–6.3% began in the stretched zone, i.e., when the reinforcement reached its yield strength. In the over-reinforced beams (µ = 8.4%) the failure is brittle, i.e., it occurs in the compressed zone [39].

The failure of ACPBF beams having a percentage of longitudinal reinforcement equal to 0.78–6.3% began in the tensile zone, i.e., when the reinforcement reached its yield strength. In addition, in the over-reinforced beams (µ = 8.4%) the failure is brittle, i.e., it occurred in the compressed zone. That is, the failure of ACPBF beams is similar to that of ACPBF beams with the addition of fibers. In the tested beams (having a percentage of longitudinal reinforcement of 6.3%), relative strains in the outermost compressed fibers of the rubber and reinforcement reach the limit values almost simultaneously [40].

**Figure 11:**
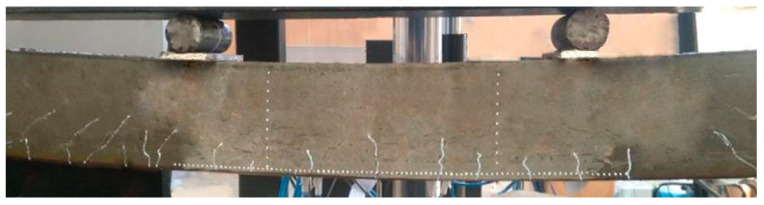
Fracture along the compression zone of the CPS beam.

The failure of ACPBF beams having the zone reinforcement, i.e., possessing 3/4 of the cross-sectional height and longitudinal reinforcement in the range of 0.8–4.95% occurred when the reinforcement reached its yield strength, i.e., in the tensile zone. In the over-reinforced beams (µ = 6.4%) the failure is brittle in nature, i.e., it occurred in the compressed zone. Consequently, to increase the bearing capacity of ACPBF beams having 3/4 of the cross-sectional height with a percentage of longitudinal reinforcement greater than 4.95%, it is necessary to reinforce the compressed zone. The earlier destruction of the ACPBF elements with zone reinforcement in the compressed zone is conditioned by the fact that the strength of the stretched zone corresponds to the solid ACPBF elements. At the same time, the strength of the compressed zone is lower than that of ACPBF elements, because the ultimate strength of non-dispersion reinforced ACPBF in compression is lower than that of ACPBF with the fiber. This leads to the fact that due to the higher tensile strength of ACPBF, the crack height develops and, consequently, the position of the neutral axis shifts towards the outermost compressed fiber, and the compressed zone is fractured (Figure 11).

The magnitude of the destructive bending moment during destruction along the compressed zone changes insignificantly. Due to the fact that the deformation of the reinforcing bar does not reach the yield strength compared to the previous series of beams, it, as a result, leads to the curvature of the graph “M_u_-µ”. It is worth noting that the increase in the difference of bending moment values for CPB and ACPB beams with fiberglass, with µ = 6.3% is due to the fact that in CPB beams the beginning of reinforcement flow almost coincided with the material reaching its yield strength in the compressed zone, but occurred slightly earlier [41].

To compare the load-carrying capacity of ACPB bending elements with longitudinal reinforcement having the load-carrying capacity of reinforced concrete elements, beams containing the B25 class concrete with different reinforcement contents were tested [42].

The results of the pure bending test of reinforced concrete beams are summarized in Table 4.

It is worth noting that ACPB beams (having a 3.55% longitudinal reinforcement) failed in the tensile zone. Reinforced concrete beams with the same content as the longitudinal reinforcement failed in the compressed zone [43].

As a result of the experiments, we can say the following:

1. The normal sections of ACPBF bending elements strength exceeds the strength of reinforced concrete elements having µ = 0.8% by 70.56%, µ = 1.25% by 44.8%, µ = 1.8% by 36.8% and by 98.9% (for beams with µ = 3.55%).

2. The normal sections of the durability of the ACPB bending elements is higher than the durability of reinforced concrete elements having µ = 0.8% by 33.7%, µ = 1.25% by 21.5%, µ = 1.8% by 17.2% and by 91.2% (for beams having µ = 3.55%). The strength of normal sections of FRCP bending elements having a zone reinforcement is higher than the strength of reinforced concrete elements having µ = 0.8% by 64.6%, µ = 1.8% by 27.3%, and by 93.4% (for beams possessing µ = 3.55%).

Figure 11 for ACPB beams shows the dependence of the strength of the normal section on the fiber-height reinforcement zone. The dependence of the figure shows that the normal section strength increases along with an increase in the height in the fiber reinforcement zone. It should be considered that the influence of the percentage of longitudinal reinforcement is more significant than the influence of this factor. The fracture along the stretched zone most fully shows the increase in the strength of the normal sections of the CPB. The percent change in longitudinal reinforcement varies with the effect of the fiber reinforcement zone on strength. An increase in the height for the fiber reinforcement zone of the CPB from zero to 120 mm gives a strength increase in the elements by 28% (percentage of longitudinal reinforcement of µ = 0.8%) and 14% (bent elements of µ = 8.4%). In this case, the strength of the normal sections practically does not change.

The bearing capacity of the bending elements with fiber reinforcement in the tensile zone and with fiber reinforcement throughout the cross-section height is higher than that of similar ACPB (without dispersion reinforcement) bending elements [44]. This is due to the fact that the magnitude of reinforcement anchorage in FKPB is higher than that in CPB (i.e., more reliable joint work). This prevents a sharper development of plastic deformations in reinforcement. In addition, disperse reinforcement increases the duration of joint work of reinforcement and polymer concrete of the stretched zone, thereby “postponing” the moment of redistribution of stresses arising in the stretched zone to the reinforcement bar, even in the sections with cracks formed. That is, this happens in the places where there are no cracks, i.e., in the places where the polymer concrete itself is absent, the metal cord fibers continue to resist tension [45].

The bearing capacity of polymer concrete bendable elements is higher than that of similar concrete elements with longitudinal reinforcement. It is related to a number of factors: a part of polymer concrete of the stretched zone above the formed crack participates in the work of normal sections; high adhesion of reinforcement and structural material (CPB, FCPB) prevents sudden development of plastic deformations in reinforcement; higher strength of the compressed zone material. We cannot exclude the fact that the material of the structure in the gap between the cracks takes part in the work of the element as a whole.

It should be noted that CPB is a more plastic material, which is confirmed by the deformation diagrams in Figure 6 and Figure 7 than heavy concrete is. It allows it to work better in tension, and therefore increases the duration of joint work of longitudinal reinforcement bars with polymer concrete before the crack formation, the introduction of steel cord fibers in the mixture further increases these figures, which can also be observed in the deformation diagrams shown in Figure 6 and Figure 7.

### 3.3. Finite Element Model of Beams Made of Polymer Concrete on a Rubber Binder Implemented in the Ansys Environment

**Figure 12:**
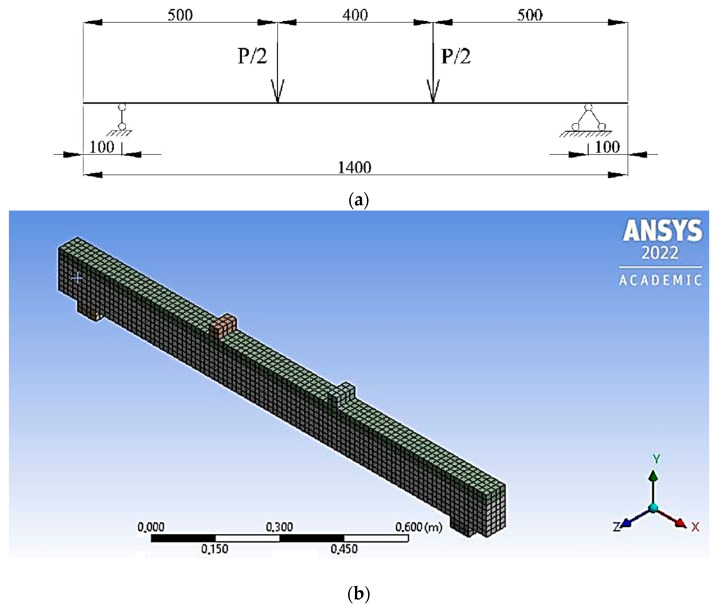
Calculation scheme of the element under study (**a**) and Finite element model of the element under study (**b**).

In order to verify the theoretical conclusions and evaluate the results of the experiment of polymer concrete beams, finite element modeling was implemented in the Ansys software package (PC), taking into account the nonlinear properties of materials. Articulated boundary conditions are set on the right support of the beam, prohibiting only vertical movements, but allowing all rotations. Hinged-fixed boundary conditions are set on the left support. The span between the centers of the supports is 1.2 m. The length of the support zone was 60 mm, and the length of the beam overhangs was 70 mm. Two concentrated forces act on the beam, mirrored in the same way as the test scheme (Figure 12).

In the Ansys environment, as an element simulating the concrete body of the sample, an eight-node finite element Solid 65 was chosen, which has the ability to simulate plastic deformations, cracks, and destruction. This finite element implements the Willam-Warnke concrete deformation model. This dependence is an ellipse equation describing the deviatoric section.

When specified in a PC, the model includes the following parameters: stress-strain diagram for compression, modulus of elasticity, ultimate compressive and tensile strength, Poisson’s ratio, and shear force transfer coefficient for closed and open cracks.

For modeling longitudinal and transverse reinforcement, the Beam 188 finite element was used, which is a rod, spatial finite element.

**Figure 13:**
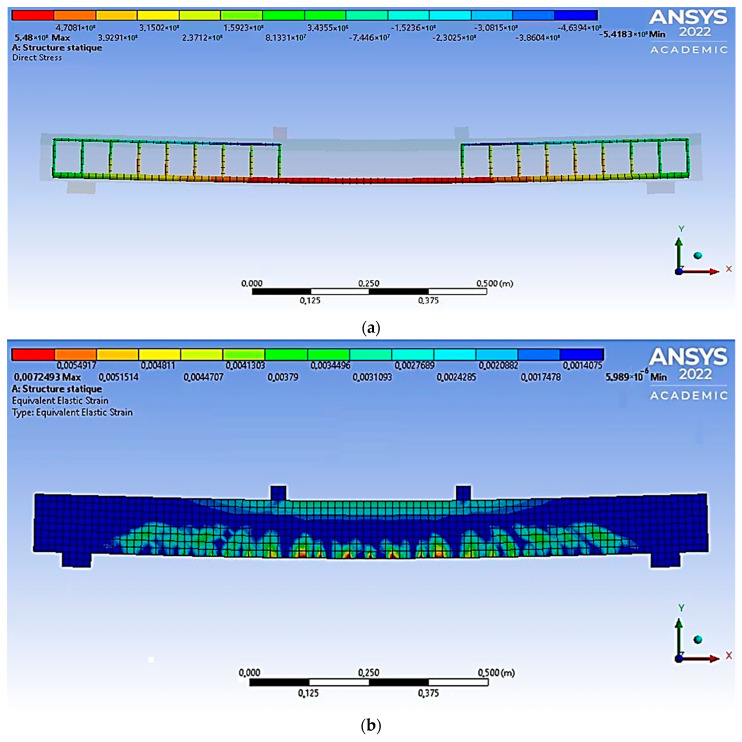
Normal stresses in reinforcement along the abscissa axis in the FE model of a beam (**a**) and inelastic deformations in the simulation model of the ACPBF beam during destruction (reinforcement percentage of 6.3) (**b**).

Based on the results of the calculation, the values of the destructive bending moments were obtained. The results of numerical studies of armocouton beams in the Ansys environment are graphically presented in Figure 13.

Simulation of the state of the beam before its destruction shows the general logic of the destruction of bending elements. This logic is consistent with the standard model, which observes the cracks’ appearance and their subsequent development.

The study results of concrete strength control (CSC) of bendable elements are summarized in Appendix A.2.

**Figure 14:**
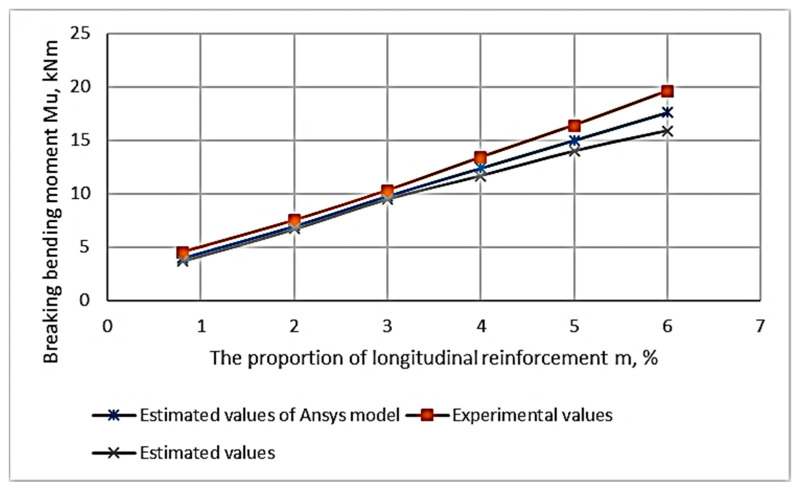
Dependence of the ultimate bending moment on the percentage of reinforcement in ACPB beams.

**Figure 15:**
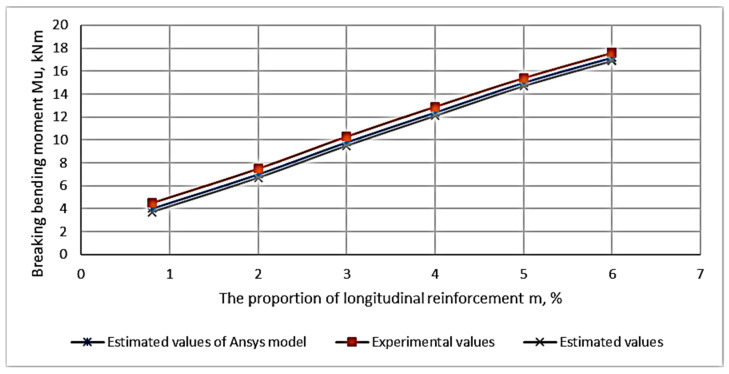
Dependence of the ultimate bending moment on the percentage of reinforcement in ACPBF beams.

The dependence of the ultimate bending moment on the percentage of longitudinal reinforcement for CPB-reinforced beams is shown in Figure 14; Figure 15 shows CPB-reinforced beams having fibers.

The Willam Warnke strength theory developed for composite materials (implemented in this work with the help of Ansys) allows the calculation of ACPB beams. The performed calculations and their comparison with experimental data showed that the maximum discrepancy for the strength values obtained by empirical and calculated methods was 14% (in the series of BPC beams). In addition, a similar deviation of 7.4% for BPC-8 beams was already obtained. The greatest discrepancy between the results was achieved for ACPB BPC beams and amounted to 18.0%.

That in turn will allow the conducted experimental studies to replace the numerical ones, but in conditions of non-compliance with the design requirements or deviation from the norms in the tests, these features should be additionally taken into account in the modeling.

## 4. Conclusions

1. The features of the stress-strain state of normal sections of ACPB beams are revealed. It has been established that the tensile strength in axial compression and tension, deformations corresponding to the tensile strength for CPB, exceed the similar parameters for the used cement concrete 2.5–6.5 times. According to the results of the work, it was found that the addition of fiber reinforcement increased the tensile strength of the BPS 3.0–7.5 times.

2. The percentage of longitudinal reinforcement and the height of the fiber reinforcement zone are the main factors influencing the strength and crack resistance of normal sections of the ACPB bending elements. The destruction of ACPB and ACPBF bending elements with a percentage of longitudinal reinforcement of less than 6.3% occurs in the tension zone, and with a percentage of longitudinal reinforcement of more than 6.3%, the destruction occurs in the compressed zone.

3. Increasing the height of the fiber reinforcement zone of the CPB from 0 mm to 120 mm, the strength of normal sections of bending elements with a percentage of longitudinal reinforcement µ = 0.8% increases by 28%. The strength of normal sections of elements with µ = 6.3% increases by 14%, for elements having µ = 8.4% the strength of normal sections practically does not increase. It has been established that fiber reinforcement increases the moment of formation of cracks in structures up to 1.5 times, and the bearing capacity of structures as a whole increase up to 1.3 times.

4. It has been experimentally determined that the addition of fiber over the entire height of the element section with the same percentage of longitudinal reinforcement increases the moment of cracking of the ACPBF of the bent elements compared to the elements from the CPB (without dispersed reinforcement) up to 1.5 times. It can be compared to the ACPBF elements with zone reinforcement (with fiber reinforcement at 3/4 of the section height) of up to 1.08 times.

5. Ultimate deflections of ACPB bending elements are less than deflections of ACPBF bending elements with zone reinforcement of up to 18.5%. Deflections of ACPB beams with a percentage of longitudinal reinforcement µ ≤ 1.8% are similar to reinforced concrete bending elements made of heavy cement concrete.

## Figures and Tables

**Table 1:** Main characteristics of used rubber polymer concrete.

Properties	Indicators for Rubber Concrete Based on Rubber Grades	
	Nitrile Butadiene Rubber (NBR)	Cis-Butadiene Low Molecular Weight Rubber (CBLMW-R)
Compressive strength, MPa	60–110	76.9–100.3
Tensile strength, MPa	8–20.0	13–18
Modulus of elasticity, MPa	(2.0–3.5) ×10^4^	(1.5–1.8) × 10^4^
Compression duration factor	0.77–0.78	0.72–0.76
Poisson’s ratio	0.18–0.35	0.2–0.3
Heat resistance, °C	90–100	100–110
Freeze resistance, many cycles of a thawing and freezing process	500	500
Abrasibility, g/cm^2^	0.15–0.30	0.25–0.79
Water suction, wt. %	0.05	0.05
Reduction, mm/m	0.17–0.21	-

**Table 2:** Physical properties of sand and crushed stone.

Filler	Size of Fractions, mm	Bulk Density, g/cm^3^	Raft Density, g/cm^3^	Specific Surface Area, cm/g^2^	Hollowness, %
Granite rubble	5.00–10.00	1.50	2.67	5.4	41.4
Quartz sand	1.25–2.50	1.61	2.65	33.0	39.1
	0.63–1.25				
	0.32–0.63				

**Table 3:** Composition of rubber fiber-polymer concrete.

Name	Component Content, wt. %
Quartz sand	24.2
CBLMW-R low molecular weight rubber	8.2
Ashes	7.8
Sulfur technical	4.0
Zinc oxide	1.2
Tiuram-D	0.4
Metal cord fibers (fiber)	2.5
Calcium oxide	0.4
Granite rubble	The rest (51.3)

**Table 4:** Test results of reinforced concrete bendable elements.

Girder Sample Code	Percentage of Longitudinal Reinforcement µ, %	Destruction Zone	Breaking Bending Moment M_u_, kNm
PC 001	0.80	Stretched	2.23
PC 002	1.25	Stretched	3.55
PC 003	1.80	Stretched	4.99
PC 004	3.55	Compressed	5.65

## Data Availability

The data presented in this study are available from the corresponding authors upon reasonable request.

## Appendix A. Results of Experimental Investigations for Breaking Bending Moments of Beam Samples

### Appendix A.1. Results of Experimental Investigations for Breaking Bending Moments of Beam Samples

This appendix updates the results of experiments obtained for the breaking bending moment of beam specimens as a function of the proportion of longitudinal type reinforcement and the height of the fiber reinforcement area, which are presented in Table A1.

**Table A1:** Values of breaking bending moments obtained from the experiment.

Girder Type	Percentage of Longitudinal of Reinforcement µ, %	Fibre Layer Height Reinforcement hf, mm	Destruction Zone	Destructive Bending Mu Moment, kNm
FPC 001	0.00	125	Stretched	2.4
FPC 002	0.80	125	Stretched	3.67
FPC 003	1.25	125	Stretched	5.21
FPC 004	1.60	125	Stretched	6.28
FPC 005	1.80	125	Stretched	6.68
FPC 006	2.5	125	Stretched	8.75
FPC 007	3.55	125	Stretched	11.31
FPC 008	4.95	125	Stretched	15.09
FPC 009	6.3	125	Stretched	17.48
FPC 010	8.4	125	Compressed	16.69
FPC 011	0.00	95	Stretched	2.28
FPC 012	0.80	95	Stretched	3.71
FPC 013	1.80	95	Stretched	6.29
FPC 014	3.55	95	Stretched	10.92
FPC 015	4.95	95	Stretched	14.78
FPC 016	6.4	95	Compressed	16.39
PC 001	0.00	0	Stretched	1.88
PC 002	0.80	0	Stretched	3.02
PC 003	1.25	0	Stretched	4.31
PC 004	1.80	0	Stretched	5.92
PC 005	2.5	0	Stretched	7.91
PC 006	3.55	0	Stretched	10.29
PC 007	4.95	0	Stretched	14.11
PC 008	6.3	0	Stretched	15.41
PC 009	8.4	0	Compressed	15.91

### Appendix A.2. Experimental Results for Breaking Bending Moments of Beam Samples

This section provides data on the bending moments of beam specimens leading to failure. Based on these data, a beam failure model was formed. Thus, the model shows the appearance and development of more areas of inelastic deformation (cracks). The results of the theoretical experimental and numerical studies of concrete strength control (CBC) of bending members are summarized in Table A2.

**Table A2:** Results of the studies conducted.

Girder Code	µ, %	Mut, KnM	Mue, KnM	Mum, KnM	∆Mum, %	∆Mut, %
PC 001	0.8	2.51	2.96	2.6	−14.0	−18.0
PC 002	1.25	4.02	4.28	4.2	−1.9	−6.5
PC 003	1.8	5.82	5.8	6.0	3.3	0.3
PC 004	2.5	7.92	7.82	8.0	2.3	1.3
PC 005	3.6	10.86	10.3	11.2	8.0	5.2
PC 006	4.95	14.47	14.0	15.2	7.9	3.2
PC 007	6.3	14.96	15.32	16.8	8.8	−2.4
FPC 001	0.8	4.00	3.78	3.52	−7.4	5.5
FPC 002	1.25	5.52	5.10	5.16	1.2	7.6
FPC 003	1.8	7.39	6.77	6.8	0.4	8.4
FPC 004	2.5	9.85	8.77	8.8	0.3	10.9
FPC 005	3.6	12.63	11.28	12.0	6.0	10.7
FPC 006	4.95	16.64	15.10	15.4	1.9	9.3
FPC 007	6.3	17.49	17.52	17.72	1.1	−0.2

Note: Mut—destructive bending moment according to the results of calculation by the proposed method; Mue—destructive bending moment according to the test results; Mum—destructive bending moment according to the results of calculation in PC “Ansys”; ∆Mum—deviation of calculation results according to the proposed method from experimental values; ∆Mut—deviation of calculation results in PC “Ansys” from experimental values.
